# Chicken heterophils as evolutionary models: insights into myeloperoxidase-independent antimicrobial strategies

**DOI:** 10.3389/fimmu.2026.1931366

**Published:** 2026-08-03

**Authors:** Seung Je Woo, Thirubasyini Songodan, Minseok Seo, Jae Yong Han

**Affiliations:** 1 Department of Agricultural Biotechnology and Research Institute of Agriculture and Life Sciences, Seoul National University, Seoul, Republic of Korea; 2 Department of Computer Science and Software Engineering, Korea University, Sejong, Republic of Korea

**Keywords:** chicken, genome editing, heterophil, myeloperoxidase, neutrophil, single cell RNA sequencing

## Abstract

The study of granulocytic phagocytes has been dominated by mammalian neutrophils, creating significant knowledge gaps in understanding functionally analogous granulocytes (heterophils) across vertebrate evolution. This review systematically examines chicken heterophils, comparing their biology to mammalian neutrophils to elucidate conserved and divergent molecular programs. Heterophils represent functionally analogous but mechanistically distinct antimicrobial effector cells characterized by fusiform granules and absence of myeloperoxidase (MPO) activity. Despite lacking MPO-dependent oxidative mechanisms, heterophils employ sophisticated non-oxidative strategies including cationic antimicrobial peptides, lysozyme, and heterophil extracellular traps (HETs). Transcriptional programs governing granulopoiesis show remarkable conservation across vertebrates, with C/EBP family transcription factors and G-CSF signaling maintaining core regulatory functions. However, comprehensive cross-species comparison between heterophils and neutrophils remains challenging, as molecular characterization of heterophil maturation and inflammatory programs has been difficult due to low recruitment numbers in conventional models. Genetically engineered chicken models now provide unprecedented opportunities for high-resolution analysis of heterophil subset diversity and inflammatory gene programs due to expanded myeloid populations. These advances position heterophils as ideal models for dissecting MPO-independent antimicrobial mechanisms and understanding primitive regulatory programs inherited from divergent comparator immune systems.

## 1 Introduction

The study of granulocytic phagocytes has been dominated by mammalian neutrophils, with the majority of mechanistic insights derived from human and murine systems. While this approach has revealed critical functions including neutrophil extracellular trap (NET) formation and diverse antimicrobial mechanisms (1), it has created significant knowledge gaps in our understanding of functionally analogous granulocytes across vertebrate evolution. The traditional paradigm of extrapolating mammalian neutrophil findings to all vertebrate granulocytes is increasingly recognized as inadequate, particularly as emerging diseases affect non-mammalian vertebrates and biomedical research increasingly relies on diverse model organisms (2). Additionally, the comparative analysis of mammalian and non-mammalian granulocytes has revealed fundamental insights into conserved inflammatory pathways that are crucial for developing treatments for granulocyte-mediated diseases (3–5).

Heterophils are predominant granulocytic phagocytes in non-mammalian vertebrates such as several birds, reptiles, amphibians and fish, functionally analogous to mammalian neutrophils (6). Avian heterophils are characterized by fusiform granules and brick-red appearance with Romanowsky stains, yet they fundamentally differ from mammalian neutrophils in their absence of myeloperoxidase (MPO) activity. Instead, avian heterophils rely on alternative antimicrobial mechanisms, including acid phosphatase, and various non-lysosomal enzymes housed within their characteristic cytoplasmic granules (6). Despite the absence of functional MPO activity, chicken heterophils maintain a well-established NADPH oxidase (NOX2) system, producing reactive oxygen species (ROS), albeit with attenuated level than neutrophils (7).

Vertebrate granulocyte evolution reflects complex interactions between conserved antimicrobial functions and lineage-specific adaptations. It was recently reported that zebrafish, human, and mouse neutrophils share highly conserved maturation and inflammatory gene programs (8, 9). Another conserved feature includes the emergence of colony-stimulating factor (CSF) receptor/ligand parings, particularly CSF3R/CSF3 evolution after jawed fish divergence, which fundamentally reorganized granulocyte distributions (3). However, fundamental questions remain regarding the extent to which avian heterophils share conserved versus divergent molecular programs with neutrophils, primarily because comprehensive knowledge of heterophil maturation trajectories and inflammatory gene signatures has been lacking (10, 11). The complete absence of MPO activity in avian heterophils versus its central role in mammalian neutrophils exemplifies how evolution has generated mechanistically distinct yet functionally equivalent antimicrobial strategies (6).

This review systematically examined chicken heterophil biology-the most extensively studied non-mammalian granulocyte-with particular emphasis on their ontogeny, distinctive morphology, functional heterogeneity, as well as their evolutionarily divergent features compared to mammalian neutrophils (mostly in humans and mice). Then, we documented heterophils serve as an ideal model for dissecting the functional capacity of MPO-independent granulocytes and providing insights into primitive regulatory programs inherited from common ancestral immune systems through cross-species analyses. Finally, we present future perspectives highlighting novel opportunity to investigate heterogenous reprogrammed heterophils in genetically engineered chicken models, enabling unprecedented high-resolution analysis of heterophil subset diversity, maturation, and inflammatory gene programs.

## 2 Overview of chicken heterophils

### 2.1 Morphological characteristics

The term “heterophil” was originally coined to describe avian granulocytes that exhibit morphological and staining characteristics distinct from mammalian neutrophils, reflecting their “different” (hetero-) affinity (-phil) for histological dyes (12). Avian heterophils are characteristically round cells measuring approximately 10-15 μm in diameter that, when stained with Romanowsky dyes, display distinctive brick-red cytoplasmic granules with fusiform (spindle-shaped) morphology (13). These primary granules frequently contain a central refractile body that may be proteinaceous in nature, distinguishing them morphologically from the round granules typical of mammalian neutrophils (14, 15). The mature heterophil nucleus is typically lobed with fewer nuclear segments than mammalian neutrophils, usually containing 2–3 lobes connected by thin chromatin strands, and exhibits heavy chromatin clumping characteristic of the species. Most avian species also possess a secondary type of smaller, round granule that appears less dense under electron microscopy and may represent a distinct functional compartment. The cytoplasm of normal mature heterophils is colorless and non-vacuolated, contrasting with the basophilic cytoplasm observed in immature forms during development (6, 13).

### 2.2 Gene marker for isolating chicken heterophils

While human neutrophil markers are firmly established for their isolation, the identification of chicken heterophils presents unique challenges due to the limited availability of species-specific surface markers (**Table 1**). Currently, no specific flow cytometry-applicable surface markers for granulocyte subsets are definitively established in chickens, necessitating alternative identification strategies. Heterophils can be operationally defined as cells lacking expression of B-cell markers (Bu-1), T-cell markers (CD3, CD4, CD8α, TCRαβ, TCRγδ), macrophage markers (MRC1L-B), and thrombocyte markers while maintaining high side scatter (SSC) characteristics indicative of their granular cytoplasm (16, 17). Given the context- and stage-dependent overlap of macrophage and neutrophil marker expression, it is particularly important to define and validate heterophil-specific markers that reliably distinguish heterophils from macrophages across developmental and inflammatory states (18). Sekelova and colleagues revealed heterophil-specific protein markers such as MMP9, SERPINB1, potentially used as pan-heterophil markers (19). Monoclonal antibodies anti-GRL1 and -GRL2 stain the granules of chicken granulocytes and thrombocytes, with increased expression detectable after cell permeabilization for intracellular staining. Nevertheless, surface expression of GRL2 can also be found on activated T cells, limiting its specificity for heterophil identification (20). The antimicrobial peptide CATH-2 can be detected using rabbit polyclonal serum through intracellular staining, providing another potential marker for heterophil identification (21).

**Table 1:** Comparison of functions and maturation between heterophils and neutrophils.

	Chicken heterophil	Human neutrophil
Maturation		
Markers	Limited, MMP9, EXFABP, CTSG, SERPINB1, GRL1, GRL2.	CD66b, CD16, CD11b, CD15, CD33, CD10 (maturation marker).
Transcription factors	NF-M (eosinophilic differentiation), PU.1 (myeloid differentiation), c-Myb (early granulopoiesis). Transcription factors essential for neutrophils are potentially conserved, but need further studies.	C/EBPα (Early), C/EBPβ (Mid, emergency granulopoiesis), C/EBPϵ (Terminal), PU.1 (dose-dependency), GFI1 (repress monocyte commitment).
Cytokines	G-CSF (granulocyte proliferation), GM-CSF (myeloid progenitor proliferation). Cytokines for heterophil maturation need further studies.	G-CSF (most critical for commitment), GM-CSF (early myeloid precursor).
Functions		
Oxidative burst	Reduced due to the absence of MPO activity. Tightly regulated by superoxide dismutase (SOD), catalase (CAT), and glutathione peroxidase (GPX).	High due to MPO activity.
Phagocytosis	Primarily non-oxidative phagocytosis.	Both oxidative and non-oxidative phagocytosis.
Degranulation	Non-oxidative antimicrobial components, including cathelicidins (CATH1, CATH2, CATH3), avian β-defensins (AvBD1-10), lysozymes, cathepsins (CSTA, CSTB, CTSC), and metalloproteinase (MMP9).	Azurophilic: MPO, Neutrophil elastase Specific: Lactoferrin, NGAL Gelatinase: MMP9, lysozyme Secretory: Fc receptors, adhesion molecules
Extracellular traps	NOX-independent heterophil extracellular trap (HET) formation by PAD (isotype not determined) and P2X1 receptor signaling. NOX-dependent HET formation	NOX-independent Ca^2+^, mitochondrial ROS-mediated neutrophil extracellular trap (NET) formation by PADI4. NOX-dependent NET formation.

### 2.3 Development and maturation of chicken heterophils

In mammals, the developmental trajectory of neutrophils is well-defined and tightly regulated. The process initiates in the bone marrow, where multipotent hematopoietic stem cells (HSCs) differentiate into common myeloid progenitors (CMPs) and subsequently into granulocyte-monocyte progenitors (GMPs) (22). Commitment to the granulocytic lineage yields myeloblasts, which then advance through consecutive morphological stages—promyelocyte, myelocyte, and metamyelocyte—before maturing into functional neutrophils (23). This progression is orchestrated by a specific network of transcription factors and cytokines. For instance, CCAAT/enhancer-binding protein α (C/EBPα) regulates the early CMP-to-GMP transition (24), C/EBPϵ directs terminal granulocyte maturation (25), and C/EBPβ mediates rapid neutrophil expansion during emergency granulopoiesis (26). Lineage bifurcation is governed by PU.1, where low expression permits granulocytic commitment and high expression induces a monocyte fate (27). Antagonistic regulators further refine this process: GFI1 represses monocytic genes (e.g., *CSF1*, *ERG-2*) to favor granulopoiesis (28), whereas IRF8 represses granulocyte fate to promote monocyte and dendritic cell development (29). Alongside these transcription factors, granulocyte colony-stimulating factor (G-CSF) serves as the primary cytokine required for steady-state neutrophil differentiation and ultimately triggers the release of mature neutrophils into peripheral blood by altering chemokine gradients (30, 31).

Although the molecular mechanisms governing chicken heterophil maturation are not as comprehensively mapped, the fundamental cellular stages and key molecular players are present in chicken as well. For instance, the PU.1 is expressed in chicken myeloid cells, binds the macrophage *CSF1R* promoter, and drives the myelomonocytic differentiation pathway (32), although it remains to be explored whether low expression of PU.1 supports heterophil development as shown in mammals. Furthermore, NF-M (the avian homolog of C/EBPβ) drives granulocytic differentiation in multipotent avian hematopoietic progenitors (33). Early myeloid progenitor proliferation is heavily regulated by c-Myb, a master regulator that cooperates with NF-M to induce granulopoiesis via activation of the *mim-1* gene (34), while v-Myb, the oncogenic form of c-Myb, halts avian differentiation entirely at the myeloblast/promyelocyte stage (35). On the cytokine level, avian homologues perform equivalent roles to their mammalian counterparts: chicken G-CSF (CSF3) and GM-CSF (CSF2) drive the survival, proliferation, and differentiation of bone marrow granulocyte progenitors (36, 37). Following maturation, heterophil effector functions are primed by pro-inflammatory cytokines such as IL-2, IFN-γ, IL-6, and IL-1β (38–41), and are kept in check by immunosuppressive molecules like IL-10 and TGF-β4 (42). Therefore, while the key homologous players of granulopoiesis are distinctly present in chickens, the precise regulatory networks governing avian heterophil development remain poorly understood compared to the well-established mammalian neutrophil pathways (**Table 1**).

The maturation of chicken heterophils is an age-dependent developmental process. Although heterophils are the predominant leukocyte in the circulating blood and gastrointestinal tract of newly hatched chicks (43, 44), their initial functional capability is significantly lower than that of adult birds. Studies demonstrate that the high susceptibility of young chicks to *Salmonella* infections directly correlates with this early functional deficiency, as heterophils do not reach full functional maturity until approximately 21 days post-hatch (45). Despite this early immaturity, neonatal heterophils still provide critical baseline innate protection. For instance, *in ovo* administration of synthetic DNA containing unmethylated CpG motifs (CpG-ODN) successfully primes the chick’s innate immune system against post-hatch *Salmonella enteritidis* exposure. This indicates that immature heterophils can effectively recognize and respond to pathogens via Toll-like receptor (TLR) pathways (46), even though non-TLR-mediated activation, such as IL-2 stimulation, remains highly age-dependent (47). To compensate for delayed systemic maturity, immature heterophils migrate to the gut prior to hatching and undergo *in situ* maturation via extramedullary granulopoiesis. This localized baseline maturation is driven by beta-defensin and presenilin-1 gene expression and occurs independently of environmental stimuli. However, upon subsequent post-hatch exposure to feed and commensal bacteria, these gut-localized heterophils rapidly upregulate pro-inflammatory cytokine production, bridging the gap before adaptive immunity develops and securing essential early protection for the growing chick (44).

### 2.4 Metabolism of chicken heterophils

In neutrophils, cellular metabolism undergoes a dynamic, stage-specific reprogramming during granulopoiesis. Immature neutrophil precursors-spanning the myeloblast through myelocyte stages-depend heavily on oxidative phosphorylation (OXPHOS) and fatty acid oxidation to meet their high energy demands for proliferation and differentiation (48). This metabolic state is fueled by autophagy-mediated lipolysis of intracellular lipid droplets, which releases free fatty acids that are directed into the mitochondrial TCA cycle and OXPHOS (49). As neutrophils terminally mature, mitochondrial mass and activity decline markedly, and energy production shifts decisively from OXPHOS back to glycolysis. Consequently, mature circulating neutrophils rely predominantly on glycolysis for ATP production, and inhibition of mitochondrial respiration does not affect their ATP generation or most effector functions (48). However, mitochondria are not entirely quiescent in mature cells; recent isotope tracing and extracellular flux analysis demonstrated that upon stimulation with certain activating stimuli (e.g., ionomycin and monosodium urate crystals), the TCA cycle is rapidly re-activated in mature neutrophils to support effector functions including NET formation and migration, while mitochondrial pyruvate carrier inhibitors suppress this re-activation and impair NET release (50). Whether a similar maturation-linked metabolic shift occurs in chicken heterophils has not been directly investigated, and this remains an open question in avian immunometabolism.

Similar to mature neutrophils, mature circulating heterophils depend on glycolysis as their primary energy-generating pathway. Studies examining heterophil extracellular trap (HET) formation have demonstrated that glucose uptake and glycolytic activity are essential for this antimicrobial function. Specifically, fumonisin B1 (FB1)-induced HET release was shown to depend on glucose/MCT1 uptake and glycolysis in chicken heterophils, with key glycolytic enzymes such as hexokinase II and 6-phosphofructo-2-kinase/fructose-2,6-bisphosphatase 3 playing regulatory roles (51). Diverse stimuli-including ochratoxin A (OTA), T-2 toxin, zearalenone (ZEA), diacetoxyscirpenol (DAS), cyclopiazonic acid (CPA), sodium molybdate, and *Toxoplasma gondii*-further confirmed that HET release depends on glycolysis (52–58).

**Figure 1:**
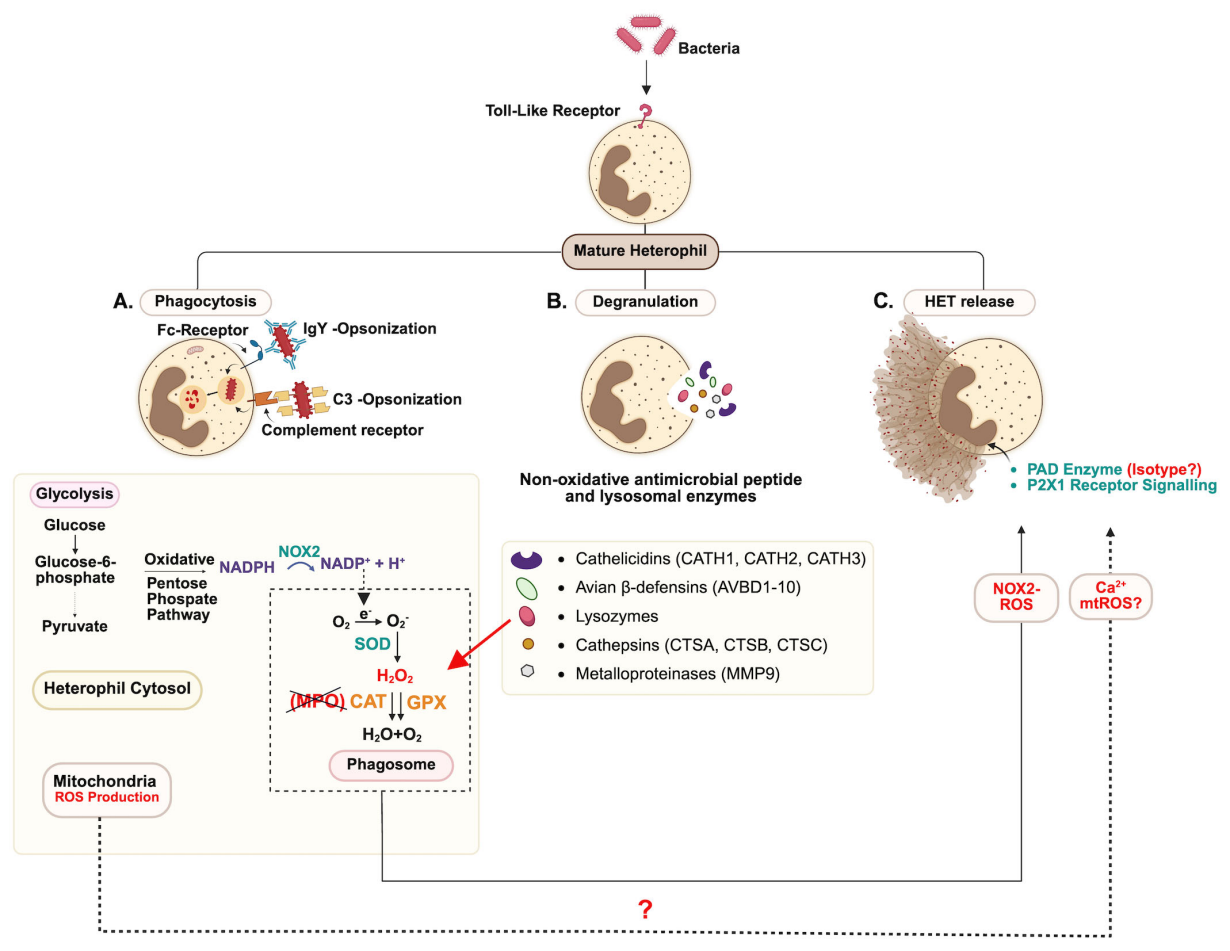
Functional features of chicken heterophils. Chicken heterophils recognize pathogens via toll-like receptors (TLRs) and execute phagocytosis, degranulation, and extracellular trap formation. Neutrophils utilize myeloperoxidase (MPO) to convert H_2_O_2_ into highly microbicidal hypochlorous acid (HOCl). In contrast, heterophils lack MPO and produce a weaker overall oxidative burst, relying on NOX2 for reactive oxygen species (ROS) generation and catalase/glutathione peroxidase (GPX) for detoxification. **(A)** Upon recognizing pathogens, heterophils generate phagosome primarily including non-oxidative antimicrobial peptides through either Fc receptor or complement receptor. **(B)** Neutrophil granules are categorized into four developmental stages (primary, secondary, tertiary, secretory), while Heterophil granules lack distinct developmental categories but compensate for reduced oxidative killing with abundant non-oxidative mediators (e.g., cathelicidins, avian β-defensins, lysozyme). **(C)** Neutrophils utilize Ca^2+^ signaling, mitochondrial ROS, and PAD4-catalyzed histone citrullination for NOX-independent NETosis, alongside classical NOX-dependent pathways, while heterophils similarly perform NOX-dependent and NOX-independent HETosis; however, due to the evolutionary absence of PADI4 in chickens, NOX-independent chromatin decondensation is likely mediated by PAD2 or PAD3.

To produce ROS, NADPH oxidase requires NADPH, which is generated by the pentose phosphate pathway (PPP)-a metabolic route that runs parallel to glycolysis, with both pathways sharing glucose-6-phosphate (G6P) as a common branching substrate (**Figure 1A**) (59). G6P is channeled either into glycolysis via phosphoglucose isomerase or into the PPP via glucose-6-phosphate dehydrogenase, the rate-limiting enzyme of the oxidative PPP. The PPP operates in two phases: an oxidative phase that generates NADPH and ribulose-5-phosphate, and a non-oxidative phase that interconverts sugar phosphates (60). During the respiratory burst, neutrophils undergo a drastic metabolic switch to a dedicated “pentose cycle” mode, in which all G6P is diverted into the oxidative PPP and the net flux through upper glycolysis is reversed to continuously recycle pentose phosphates back to G6P, thereby maximizing NADPH production to sustain NADPH oxidase activity (61).

Regarding chicken heterophils, it is important to note that NADPH oxidase dependence for HET formation is stimulus-specific and is not universal. HET release induced by OTA, T-2 toxin, ZEA, DAS, CPA, sodium molybdate, and *Toxoplasma gondii* was linked to NADPH oxidase-dependent ROS production alongside glycolysis (52–58). In contrast, FB1-induced HET formation was found to be NADPH oxidase-independent: pharmacological inhibition of NADPH oxidase with diphenyleneiodonium chloride did not reduce FB1-triggered HET release; instead, this pathway relied on PAD4 and the P2X1 receptor (62). Similarly, citrinin (CTN)-induced HETs were also NADPH oxidase-independent (63). These findings reveal mechanistic heterogeneity in HET formation and indicate that the requirement for PPP-derived NADPH in chicken heterophil function is stimulus-dependent rather than a universal feature. Supporting a broader role of the PPP in avian leukocyte immune responses, transcriptomic analysis of peripheral blood leukocytes from avian pathogenic *escherichia coli*-challenged chickens identified the PPP among enriched KEGG pathways in birds exhibiting severe pathology (64).

### 2.5 Core function of chicken heterophils

Similar to mammalian neutrophils, chicken heterophils serve as critical first-line effector cells of innate immunity, defending against pathogens through multiple antimicrobial mechanisms including phagocytosis, degranulation, and extracellular trap formation. Chicken heterophils constitutively express a broad TLR repertoire to detect diverse microbial signatures including bacterial lipoproteins, peptidoglycan, lipopolysaccharide (LPS), flagellin, double-stranded RNA, and single-stranded RNA (41). Notably, different TLR agonists activate distinct functional programs: bacterial TLR agonists (PAM3CSK4, PGN, LPS, flagellin) induce both oxidative burst and degranulation, while PAM3CSK4 alone elicits degranulation without oxidative burst. Signaling proceeds though differential p38 MAPK and ERK1/2 phosphorylation pathways, but not through JNK (41). TLR activation also drives NF-κB-dependent sequential production of leukotriene B4, followed by prostaglandin E2, representing the first documented production of eicosanoid lipid inflammatory mediators by avian heterophils (65). Additionally, TLR-stimulated heterophils upregulate pro-inflammatory cytokine mRNAs (IL-1β, IL-6, IL-8) following bacterial stimulation (41). Heterophils from *Salmonella*-resistant chicken lines produce significantly higher level of IL-6, IL-8, and IL-18 in response to *Salmonella enteritidis* challenge than heterophils from susceptible lines, indicating that heterophil cytokine output is genetically determined and linked to disease resistance (66).

The most critical biochemical distinction between avian heterophils and mammalian neutrophils is the absence of a functional MPO–H_2_O_2_–halide enzymatic system. While heterophils carry an MPO-homologous gene sequence and trace histochemical MPO activity has been detected (67), the functional MPO–halide system is non-operative, resulting in a comparatively weak oxidative response relative to mammalian neutrophils (68). In place of this, heterophils maintain an active NADPH oxidase complex that generates superoxide anion (O_2_^−^) and downstream ROS in response to PMA, LPS, and bacterial infection (69). Intracellular antioxidant enzymes-superoxide dismutase (SOD), catalase (CAT), and glutathione peroxidase (GPX)-protect the heterophil from oxidative self-damage by sequentially dismutating O_2_^−^→H_2_O_2_→H_2_O+O_2_ (70) (**Figure 1A**; **Table 1**). Recent transcriptomic studies have raised the possibility that MPO and NADPH oxidase component genes (*CYBA*, *CYBB*, *NCF2*, *NCF4*, *RAC2*) were upregulated in tissue-activated heterophils, suggesting context-dependent oxidative capacity beyond that of circulating cells (71), though this awaits functional confirmation.

Upon pathogen recognition, chicken heterophils engage a rapid phagocytic response that constitutes a critical arm of intracellular bacterial killing. Phagocytosis is triggered via two principal receptor-mediated pathways: opsonin-dependent uptake through Fc receptors (recognizing IgY-opsonized targets) and complement receptors (C3-mediated opsonization), both activating distinct but partially overlapping signal transduction cascades (72, 73). Following internalization, phagosomal killing in chicken heterophils depends predominantly on non-oxidative mechanisms-particularly cationic antimicrobial peptides and proteolytic enzymes delivered from granule fusion-due to the absence of a functional MPO–halide system (68). Despite the absence of MPO, chicken heterophils generate a detectable NADPH oxidase-dependent oxidative burst upon phagocytic stimulation. Activated by PMA, opsonized zymosan, LPS, or viable bacteria, the NOX2 complex assembles at the phagosomal membrane to transfer electrons from NADPH to molecular oxygen, generating superoxide (O_2_^−^) and downstream H_2_O_2_ (69) (**Figure 1A**). This oxidative burst is, however, approximately two orders of magnitude weaker than in MPO-containing human neutrophils and fails to completely eliminate intracellular Salmonella alone-confirming that NADPH oxidase-derived ROS in chicken heterophils is insufficient for standalone bactericidal activity (74).

In mammalian neutrophils, granule synthesis proceeds sequentially across developmental stages in the bone marrow, generating primary (azurophilic), secondary (specific), tertiary (gelatinase), and secretory granules (75). Avian heterophils likewise contain morphologically distinct granule subtypes, most notably the large rod-shaped primary granules that store Cathelicidin-2 (CATH-2); synthesis of CATH-2 is initiated at the early promyelocyte stage and the mature peptide is released upon stimulation with *Salmonella*-derived LPS through serine protease-mediated processing (21). More broadly, heterophil cytoplasmic granules are enriched in cathelicidins (CATH1, CATH2, CATH3) and avian β-defensins (AvBDs), which provide broad-spectrum bactericidal activity against both Gram-positive and Gram-negative bacteria (21, 76). Proteomics of splenic heterophils confirmed the enrichment of cathelicidin family members, lysozyme (LYZ, LYG2), and matrix metalloproteinase 9 (MMP9) as heterophil-specific markers (19). Serine proteases (elastase), cathepsins, and MMPs contribute to intracellular and extracellular antimicrobial killing (**Figure 1B**; **Table 1**) (19).

Avian heterophils form HETs-web-like extracellular DNA scaffolds decorated with antimicrobial proteins including histones and elastase-analogous to mammalian NETs (77). HET formation has been demonstrated against a broad range of stimuli *in vitro* (51, 52, 62), and against biological pathogens including *Salmonella enterica* (78), *Eimeria tenella* sporozoites (79), and most recently *Neospora caninum* (80). Heterophils trap *Salmonella* within ET structures within 15 min of exposure *in vitro*; however, live/dead staining revealed that most entrapped bacteria remain viable, suggesting that HETs may act primarily to restrict bacterial dissemination rather than directly killing *Salmonella* (78). Pathologically, excessive HET formation induced by nanoparticles (alumina Al_2_O_3_, nanosilver Ag) exacerbates hepatic and renal injury *in vivo* through ROS amplification and inflammatory cascade activation, demonstrating that dysregulated HET activity is immunopathological (81, 82).

Mechanistically, mammalian NETosis proceeds via (1) NADPH oxidase–and intragranular MPO–dependent suicidal NETosis (83) and (2) NADPH oxidase–independent vital NETosis triggered by SK3 channel-mediated Ca^2+^ influx and mitochondrial ROS (84). In chicken heterophils, NADPH oxidase-dependent ROS drives HET formation for most stimuli, while FB1- and CTN-induced HETs proceed via NADPH oxidase-independent, PAD/P2X1-dependent mechanisms (62, 63), consistent with the vital NETosis paradigm, although it needs further investigation. A key genomic constraint is that while mammalian NETosis critically requires PADI4-mediated histone citrullination (85), chickens encode only three PAD isoforms (PADI1, PADI2, PADI3) and lack PADI4 and PADI6 (86); the PAD isoform driving histone citrullination in HETs thus remains unresolved (**Figure 1C**; **Table 1**).

## 3 Heterophils as evolutionary models for therapeutic development

### 3.1 Pathological neutrophils as therapeutic targets

Under homeostatic conditions, neutrophil production is tightly regulated through steady-state granulopoiesis in the bone marrow, maintaining a constant supply of mature neutrophils. However, during systemic infections, severe inflammation, or other pathological stresses, the hematopoietic system rapidly switches from steady-state to emergency granulopoiesis-a distinct, accelerated myeloid production program designed to meet dramatically increased demand for neutrophils at sites of infection or injury (87). The primary cellular sensor driving emergency granulopoiesis is the vascular endothelium: endothelial cell-intrinsic MyD88/TLR4 signaling in response to systemic LPS or bacteremia is the dominant trigger for G-CSF production and GMP expansion, as demonstrated by endothelial-specific *Myd88* knockout mice (88). IL-1β reinforces this response by stimulating endothelial and stromal cell release of G-CSF and GM-CSF (89). Additional cytokines including IL-6 and CXCL1 act downstream of TLR engagement to amplify myeloid progenitor expansion (87). Under homeostatic conditions, the IL-23→IL-17A→G-CSF axis serves as a neutrophil rheostat-macrophages and dendritic cells secrete IL-23 to drive IL-17A production from T cells, which induces G-CSF from non-hematopoietic stromal cells; apoptotic neutrophil clearance by macrophages suppresses IL-23 to close the feedback loop (90). Emergency granulopoiesis extends beyond the bone marrow to extramedullary sites-most prominently the spleen, where SCF or CXCL12-expressing Tcf21^+^ stromal cells around perisinusoidal red pulp niches support HSC engraftment and emergency myeloid output (91).

In both acute sepsis and chronic inflammatory diseases such as rheumatoid arthritis (RA) and systemic lupus erythematosus (SLE), dysregulated emergency granulopoiesis expands neutrophil output while also generating cells with prolonged survival, heightened activation, and pathogenic tissue effects. In sepsis, neutrophil apoptosis is suppressed through Mcl-1 upregulation, PI3K/AKT-dependent survival signaling downstream of C5a and PD-L1, and impaired caspase-8 cleavage, leading to accumulation of long-lived, primed neutrophils that damage tissues through ROS, serine proteases, and NET release; these NETs further promote endothelial injury, vascular leakage, coagulation activation, and ultimately dysfunction syndrome and disseminated intravascular coagulation (92–94). A related but more persistent process occurs in RA and SLE, where chronic inflammatory cues reprogram HSPCs toward sustained myeloid bias through IL-6/JAK/STAT3, TNF-α, interferon signaling, and S100A8/A9, thereby maintaining excessive neutrophil production and inflammatory reinforcement (95–98). In RA, elevated G-CSF and GM-CSF support neutrophil expansion and synovial recruitment, where infiltrating neutrophils promote cartilage destruction through degranulation, immune complex-triggered ROS production, and NET-driven pyroptosis of synovial fibroblasts (99, 100). In SLE, low-density granulocytes and persistent, poorly degraded NETs amplify type I interferon signaling, endothelial toxicity, thromboinflammation, and glomerular injury, linking sustained emergency granulopoiesis to chronic autoimmunity, vascular dysfunction, and progressive organ damage (101–103).

These pathological roles collectively identify neutrophils and their products as actionable therapeutic targets. Current strategies include: i) PAD4 inhibition (e.g., GSK484, BB-Cl-amidine) to block histone citrullination and NETosis, reducing NET-driven immunopathology in models of thrombosis, arthritis, and lupus (104); ii) DNase I administration to degrade extracellular NET scaffolds, currently evaluated in sepsis and SLE (102); iii) CDK inhibitors (e.g., AT7519) targeting the Mcl-1 survival pathway to restore neutrophil apoptosis in sepsis-related acute respiratory distress syndrome (105); ix) G-CSF/G-CSFR blockade to reduce pathological granulopoiesis in RA and immune complex-driven arthritis (99); and v) S100A8/A9 inhibition (tasquinimod, paquinimod) to suppress extramedullary myelopoiesis and neutrophil production in stroke and chronic inflammatory contexts (106). Despite their promise, a major limitation of these current strategies is that broad suppression of MPO-dependent oxidative damage by neutrophil frequently leaves patients highly vulnerable to opportunistic infections (107). Chicken heterophil model potentially provides next-generation therapeutics that selectively dampen oxidative tissue damage without compromising the host’s primary innate defenses.

### 3.2 Conserved molecular mechanism of neutrophil across vertebrate species

To dissect mechanisms underlying neutrophil-mediated pathology, it is essential to determine how much neutrophil biology is conserved between model organisms and humans. At the transcriptional level, neutrophil maturation in zebrafish, mice, and humans is orchestrated by remarkably similar transcription factor networks. A cross-species analysis using high-resolution transcriptional profiling of transgenic zebrafish strains confirmed that immature granulocyte stages exhibit the highest molecular similarity across all three species (8), and single cell RNA sequencing (scRNA-seq) label transfer from zebrafish burn-wound neutrophils to human maturation stages further validated that three of four zebrafish neutrophil subsets map to defined human precursor, immature, and mature states (108). The hierarchical regulatory order of core C/EBP family members is conserved from teleosts to mammals (8). Additional regulators-RUNX1/CBFβ, c-Myb, and RARα, the last of which directly activates the C/EBPϵ promoter-participate in early granulopoiesis across species, demonstrating that the fundamental myeloid genetic program was established early in vertebrate evolution (109, 110).

Inflammatory signaling cascades represent another domain of substantial conservation, though critical species-specific differences must be acknowledged. Systematic transcriptomic profiling of zebrafish tissues following LPS stimulation reveals highly correlated genomic responses to those of mouse and human; approximately 75% of significantly modulated gene sets during zebrafish inflammation are also enriched in mammalian LPS-stimulated datasets (9), although LPS is distinctly sensed between zebrafish and mammals (111). The negative regulatory circuits resolving inflammation also show conservation: SOCS proteins (SOCS1, SOCS3) and their zebrafish paralogs (socs1, socs3a/b) share conserved KIR/ESS-domain-mediated JAK suppression, are induced by IL-6, TNF-α, and IFN in both fish and mammals, and fulfill equivalent negative feedback roles in limiting JAK–STAT signaling output (112). Additionally, human and mouse neutrophils share conserved inflammatory transcription programs (113), collectively indicating that neutrophil-mediated inflammation is conserved across several species.

### 3.3 Heterophils as a novel model for divergent comparator molecular mechanisms of granulocytes

Human and mouse neutrophils have been extensively characterized at single-cell resolution in diverse inflammatory contexts, enabling cross-species comparison of neutrophil gene expression programs (113–121). By contrast, single-cell transcriptomic analysis of chicken heterophils remains limited, largely because they have been captured only in low numbers in previous datasets (10, 11) (**Table 2**). Recent advances in genetic engineering have opened new avenues for addressing this bottleneck: T cell receptor β knockout chickens and Recombination activating gene 1 (*RAG1*)-deficient chicken lines exhibit significantly expanded myeloid-lineage cell populations in the spleen (123, 124), thereby providing unprecedented opportunities for in-depth scRNA-seq analysis of heterophil subsets.

**Table 2:** Single cell RNA sequencing studies for revealing molecular mechanism of their maturation and antimicrobial functions.

Neutrophil			
Studies	Species	Analyzed organs	Main findings
Nishide et al., 2025 (114)	Human	Blood	Neutrophils with type II IFN signatures is uniquely primed neutrophil subset in microscopic polyangiitis (MPA) patient.
Creusat et al., 2024 (115)	Human, Mouse	Bone marrow, lung (Mouse) Lung (Human)	When infected by highly pathogenic influenza A virus, IFN-γ remotely primes bone marrow neutrophils to express PD-L1 and acquire regulatory functions, creating a resistance/tolerance trade-off during severe viral infection.
Want et al., 2024 (116)	Human	PBMC	Neutrophil percentage is increased in adult systemic lupus erythematosus, and identified potential biomarkers (*IFITM3, ISG15, IFI16, LY6E*) for therapeutic targeting.
Hackert et al., 2023 (113)	Human, Mouse	Bone marrow, blood, air pouch, spleen (Mouse) Blood (Human)	A conserved gene core inflammation program exists across species and tissues, transcriptional regulation driven by NF-κB and AP-1 complexes, with *JunB* knockout reducing core inflammation gene expression.
Pham et al., 2023 (117)	Mouse	Spleen	*Trypanosoma brucei* infection expands splenic neutrophil populations that abnormally produce high levels of MMP-8 and MMP-9 metalloproteinases, in the absence of sufficient tissue inhibitor counterbalance, which excessively degrade the extracellular matrix and destroy the spleen’s follicular architecture needed for B cell-mediated antibody responses, ultimately impairing parasite control.
Ioannou et al., 2022 (118)	Mouse	Spleen, bone marrow (Mouse)	T cell death-derived extracellular chromatin and G-CSF selectively shorten mature neutrophil lifespan, causing shift to immature, dysfunctional neutrophils.
Zheng et al., 2022 (119)	Mouse	Lung, spleen (Mouse)	Severe COVD-19 infection caused massive neutrophil recruitment and hyperinflammatory activation, showing respiratory burst phenotype. Neutrophil-derived *NOS2* expression caused arginine exhaustion and dampened B cell function.
Wigerblad et al., 2022 (120)	Human	Blood	Analyzed differentially mature human blood neutrophils from immature, bone marrow-like neutrophil subsets to mature resting neutrophils, as well as IFN-primed neutrophil subsets expressing type I IFN-inducible genes.
Xie et al., 2020 (121)	Human, Mouse	Bone marrow, blood, spleen, liver, peritoneal cavity (Mouse) Blood (Human)	Analyzed core gene programs underlying mouse neutrophil maturation from bone marrow immature neutrophils to mature neutrophils. During *E.coli* infection, neutrophils undergo transcriptional reprogramming: immature neutrophils enhance reactive oxygen species (ROS) production, whereas mature neutrophils upregulate inflammatory cytokines and chemokines.
Heterophil			
Woo et al., 2025 (122)	Chicken	Spleen	*RAG1*-deficient spleen showed specific expansion of myeloid lineage populations, including heterophils.
Maxwell et al., 2024 (10)	Chicken	Blood	Comprehensive scRNA-seq on chicken blood identified heterophils mixed with monocytes.
Warren et al., 2023 (11)	Chicken	Spleen	Identified spleen single cell atlas between Marek’s disease virus (MDV)-susceptible and -resistant chicken line. Identified two granulocyte subsets.

Indeed, scRNA-seq has been successfully performed in *RAG1*-deficient chicken lines, revealing a marked reprogramming of myeloid cells in the spleen (**Table 2**). In this line, *RAG1* gene-which codes a core protein that complexes with RAG2 to mediate V(D)J recombination-was knocked out. This targeted disruption results in the severe failure of B and T cell development (124). Consequently, the loss of adaptive lymphocyte-mediated immune regulation triggered a hyperinflammatory environment, which in turn drove a state of emergency granulopoiesis (125, 126). These reprogrammed myeloid populations display significantly elevated expression of inflammatory genes, contributing substantially to the hyperinflammatory phenotype observed in these immunodeficient models (122). Deep molecular characterization of these reprogrammed myeloid-lineage cells, including heterophils at various maturation stages, will elucidate critical maturation trajectories and inflammatory gene signatures at the molecular level—insights that have remained a fundamental bottleneck in conducting evolutionary comparisons between heterophils and neutrophils.

Although the chicken is the primary model for heterophil biology, significant molecular and functional divergence exists across avian orders, highlighting the need to incorporate diverse species into comparative analyses. Genomically, the antimicrobial peptide repertoires within heterophil granules vary considerably across taxa. For instance, while cathelicidin genes are broadly conserved, specific subtypes are subject to lineage-specific duplication, complete loss, or pseudogenization depending on the avian order, with stark differences observed among Galliformes, Passeriformes, and Falconiformes (127, 128). These genetic variations translate directly into functional heterogeneity. Even between closely related Galliformes, such as chickens and turkeys, heterophils display divergent activation thresholds and responsiveness to specific inflammatory agonists (129). Thus, it is essential to expand single-cell and functional analyses to include phylogenetically diverse avian species and other MPO-deficient vertebrates. Such comprehensive characterization is expected to distinguish species-specific gene signatures from evolutionarily conserved molecular programs, thereby establishing strategic frameworks for addressing species-specific diseases and laying the groundwork for utilizing avian species as novel translational models for investigating neutrophil-associated pathologies.

## 4 Conclusion

The comprehensive analysis of avian heterophils presented in this review illuminates a fundamental paradigm shift in our understanding of vertebrate granulocyte biology. While decades of neutrophil research have established the MPO-dependent oxidative burst as a cornerstone of antimicrobial defense, heterophils demonstrate that evolutionarily distinct yet functionally equivalent strategies can achieve comparable pathogen clearance through sophisticated non-oxidative mechanisms. The complete absence of MPO in heterophils, coupled with their reliance on cationic antimicrobial peptides and alternative oxidative pathways, reveals the remarkable plasticity of innate immune evolution and challenges the traditional extrapolation of mammalian neutrophil findings to all vertebrate granulocytes. However, it must be acknowledged that many molecular mechanisms underlying heterophil function remain incompletely characterized, limiting our ability to fully define the extent of conservation or divergence compared to mammalian neutrophils. Despite this limitation, the conservation of core transcriptional machinery governing granulopoiesis across vertebrate evolution, combined with the unique opportunities provided by genetically engineered chicken models positions heterophils as an invaluable translational model for investigating neutrophil-associated pathologies and developing novel therapeutic interventions that target fundamental, evolutionarily conserved immune mechanisms applicable to both human medicine and agricultural applications.
